# Channels and countermeasures of the COVID-19 pandemic’s impact on urban economic resilience: Lessons from China

**DOI:** 10.1371/journal.pone.0338499

**Published:** 2025-12-12

**Authors:** Xianxiang Lu, Yangrui Duan

**Affiliations:** School of Economics, Zhongnan University of Economics and Law, Wuhan, Hubei, China; Leshan Normal University, CHINA

## Abstract

Resilience is a crucial ability of an economy to withstand sudden events and uncertain shocks. Using the entropy method, this study measures the economic resilience of 281 Chinese cities (prefecture-level and above) from 2017 to 2022, and empirically examines the impact of COVID-19 on this resilience, as well as its transmission channels. The results show that COVID-19 adversely affected overall urban economic resilience, with contrasting effects across its sub-dimensions: an insignificant negative impact on shock resistance, a significant negative impact on adaptive recovery, and an insignificant positive impact on innovative transformation. Transmission channels analysis reveals COVID-19 impaired urban economic resilience through the channels of employment structure, consumption, investment, and unrelated diversification, with consumption identified as the predominant one. Heterogeneity analysis reveals that the economic resilience of cities in both the high and low manufacturing specialization groups was more adversely affected by COVID-19 than that of cities in the medium group. Regarding services specialization, the economic resilience of cities with a medium degree of services specialization were more negatively affected by COVID-19 than that of cities with low services specialization. Furthermore, the economic resilience of cities with a higher degree of related diversification was less negatively affected by COVID-19. This study provides a replicable analytical framework and empirical evidence for enhancing urban economic resilience in China and other countries in post-pandemic era.

## 1 Introduction and literature

Economies inevitably face external shocks during development. Economic resilience refers to an economy’s ability to withstand shocks, adapt to change, and recover. This ability is crucial for economic competitiveness [1]. In the contemporary era, frequent uncertain shocks occur globally. Some economies recover rapidly and succeed in forging new growth trajectories, whereas others are often vulnerable, struggling to absorb disruptions and resume growth after shocks [2]. The observed variation in shock responses has sparked growing academic interest in economic resilience. Given the increasing uncertainties in a complex global environment, building economic resilience has become an urgent priority for economies worldwide [3]. Consequently, a critical endeavor is to dissect the influence and transmission channels of typical shock events on economic resilience.

COVID-19 meets our criteria for a typical shock event, as it is characterized by high transmissibility and elevated mortality [4], thereby directly threatening public health and disrupting socioeconomic systems worldwide. By May 5, 2023, when WHO declared that COVID-19 was no longer a global health emergency, data indicated that the pandemic had caused nearly 7 million cumulative deaths [5]. Economically, COVID-19 caused the most severe global recession since the Great Depression, with global GDP falling by 3.1% in 2020 [6]. According to the IMF World Economic Outlook database, advanced economies experienced a 4.5% contraction in 2020 (US: −3.4%, EU: −6.0%, Japan: −4.5%, Germany: −4.6%, France: −7.9%, UK: −9.3%), while emerging economies shrank by 2.0% (India: −6.6%, Russia: −2.7%, Brazil: −3.9%, South Africa: −6.4%, Indonesia: −2.1%, ASEAN-5: −3.4%, Mexico: −8.1%) [7]. Following an initial slump, China avoided a prolonged recession and posted 2.2% annual growth rate, making it the only major economy to record positive growth. This performance underscores why studying China’s economic resilience is essential for devising post-pandemic recovery strategies [8].

Given China’s vast territory and the unique developmental characteristics of its regions, cities——as fundamental units of regional socioeconomic activity——function as hubs for industrial development and other critical economic and social functions. They act as both hotspots for shock events and pivotal nodes for building economic resilience, thereby providing a granular scale for research. This study therefore adopts the city as the primary unit of analysis. First, grounded in the inherent attributes of COVID-19 and the core conceptualization of economic resilience, we constructed an indicator system to measure urban economic resilience in China. Second, we develop an empirical model to analyze the impacts of COVID-19 on the overall and sub-dimensions of urban economic resilience, and further explore the transmission channels. Finally, we conduct heterogeneity analyses across different industrial agglomeration characteristics. The study period spans from 2017 to 2022. The year 2017 was selected as the starting point because the publication of city-level data in China became more standardized commencing in this year, thereby ensuring higher data quality for constructing resilience indicators. The year 2022 was selected as the endpoint because China’s downgrading of COVID-19 management from Class A to Class B on December 27, 2022, served as a landmark policy shift that defined the end of a distinct phase of the pandemic in China. Subsequently, China scaled back the frequency and content of its pandemic reporting, leading to the discontinuation of publicly available on confirmed COVID-19 cases. The 2017−2022 period enables a comprehensive comparison of all indicators in this study between the pre-pandemic and pandemic periods, offering an appropriate time frame to examine the impact of COVID-19 on urban economic resilience.

The existing literature on the impacts of COVID-19 on economic resilience primarily adopts supply-side or demand-side perspectives in isolation. Focusing on supply-side analyses, Huang (2020) employed qualitative methods to study the effects of COVID-19, asserting that in the short run, the pandemic disrupted labor supply, enterprise production, and supply chains in China, thereby undermining economic resilience. In the long term, however, he argued that environmental changes induced by the pandemic could spur technological and institutional innovations, advance supply-side reforms, and accelerate post-crisis recovery [9]. An and Huang (2024) assessed the pandemic’s impacts through the lens of urban industrial structure, revealing that service sectors bore the brunt of the initial shocks, which subsequently propagated to manufacturing through supply chain linkages. However, urban economic recovery often initiated in service sectors as well, suggesting that a service-oriented industrial structure weakens resistance but enhances recovery capacity. The study further found that industrial diversification bolsters both resistance and recovery, whereas industrial specialization tends to produce opposing effects [10]. In contrast, Cheng et al. (2024) proposed a divergent conclusion: industrial diversification strengthens resilience in early stage of the pandemic, when economic systems face limited shocks. In the later stages, however, the intensified shock exceeded the absorptive capacity of industrial diversity, consequently undermining economic resilience [11]. Barrero et al. (2020) highlighted the pandemic as a material shock that triggered resource misallocation, noting that 32%−42% of temporary business closures in the U.S. thereby became permanent layoff. To mitigate such resource misallocation and its adverse effects on economic resilience, policies such as reducing employment barriers and providing targeted government subsidies are essential [12].

**Fig 1:**
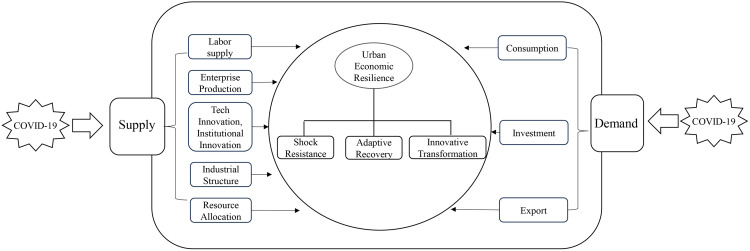
The impact of COVID-19 on urban economic resilience.

In demand-side studies, Zhou et al. (2020) examined COVID-19’s impact on China’s economy using 2017 input-output data, identifying three key transmission channels [13]. Regarding consumption, the overall propensity to consume declined, and its structure shifted toward medical supplies, while sectors like tourism, dining, retail, transport, and entertainment experienced sharp declines. For investment, the impact manifested primarily as a rising risk premium. The pandemic increased investment risks, causing investors to demand higher returns and adopt a more cautious approach to decision-making. As for exports, overseas orders in China diminished due to logistics disruptions and complex trade procedures. On the whole, domestic demand (investment and consumption) in China suffered greater damage than international demand (exports). Li and Wu (2020) analyzed household survey data from Beijing-Tianjin-Hebei region, collected between February 17 and 24, 2020 [14]. They examined the impact and transmission channels of COVID-19 on consumption from a micro perspective. Due to the value-eroding and risky nature of COVID-19, it fundamentally reduced consumer spending through three psychological channels: heightened precautionary savings, lowered income expectations, and a trend toward more deliberate consumption. Wang et al. (2024) emphasized that enterprises are the foundation for restoring normal economic order and vitality. Investments in these entities constitute a major component of total social investment, which in turn influences economic recovery and stability. However, COVID-19 exacerbated problems of information asymmetry and principal-agent conflicts. These issues constrained investments in enterprises, raised loan issuance costs, reduced capital allocation efficiency, and ultimately impeded economic system’s recovery from the shock [15]. He et al. (2023) conceptualized export resilience as a component of regional economic resilience. Using provincial data from 2020 to 2021, he analyzed the impact of COVID-19 on China’s regional export resilience from both internal and external perspectives [16]. Internally, regional export disparities were driven by three key factors: local pandemic severity, the stringency of provincial epidemic prevention policies, and variations in healthcare capacity. Externally, the foreign trade performance and economic stability of Chinese provinces were primarily shaped by the pandemic severity in their largest export destinations and the numbers of their trading partners. To clarify the potential impacts of COVID-19 on urban economic resilience, we have developed Fig 1, which integrates findings from the existing literature.

Existing studies have assessed COVID-19’s impacts on economic resilience across nations, yet they predominantly rely on qualitative methods, focus on short-term effects around 2020, lack city-level quantitative analyses, examine unilateral (supply or demand) impact channels, and overlook the role of crisis attributes in shaping economic resilience [17]. Our study makes three key contributions. First, we propose a framework for assessing economic resilience that accounts for its dynamic properties and shock characteristics, applying it to quantify COVID-19’s impacts on urban economic resilience and its sub-dimensions. Second, leveraging a relatively complete time frame, we uncover and verify the multifaceted transmission channels of COVID-19 on urban economic resilience from the perspectives of demand, supply, and industrial dynamics. Third, we examine the heterogeneous effects of COVID-19 on urban economic resilience by conducting a subgroup analysis based on industrial structure.

Nowadays, the international environment is still complicated. Economic resilience has become both a fundamental prerequisite and core competitivity for the sustainable development of cities. From the point of public health emergency, this study investigates how Chinese cities cope with typical shock, based on the agentic decision-making of economic entities and industrial structural characteristics. It seeks to develop an analytical framework applicable to the economic resilience of any city or region, and to explore feasible pathways through which cities or regions can proactively address challenges. The subsequent sections of this study are structured as follows. Section II presents theoretical background and research hypotheses. Section III outlines the methodology. Section IV reports empirical results and analysis. Section V provides discussion. Section VI states the main conclusions.

## 2 Theoretical background and research hypotheses

### 2.1 The definition and measurement of economic resilience

The research about the concept of economic resilience has evolved continuously. Even before its incorporation into economics, the conceptual framework of resilience had undergone a paradigm shift, transitioning from a focus on engineering resilience to ecological resilience and ultimately to adaptive resilience within complex systems. Engineering resilience refers to a system’s capacity to absorb external shocks while maintaining a single equilibrium state. This framework is a form of static analysis that emphasizes the speed and degree to which a system returns to its pre-shock equilibrium after a disturbance. Holling (1973) introduced the concept of resilience into system ecology and expanded its definition through comparative static analysis. He proposed that ecosystem may exhibit alternative stable states: Following a disturbance, the system may either return to its original equilibrium state, or it absorbs the perturbation while sustaining relative equilibrium among its internal subsystems [18]. In other words, ecologically resilient systems do not necessarily return to their original states after a shock, they may instead transition toward new stable states. Within this framework, resilience is characterized by the magnitude of shocks the system can absorb prior before undergoing a structural transformation. Whereas engineering resilience mainly focused on efficiency, ecological resilience began to emphasize system functionality and sustainable development. In subsequent studies, Gunderson and Holling (2002) developed the Panarchy Model, which is based on complex system theory and the concept of adaptive cycle, to describe nested, adaptive cyclical processes across different scales [19]. They posited that resilience does not require a stable equilibrium but evolves dynamically with periodic system transformations and environmental changes. This perspective represented the embryonic form of economic resilience theory. Building on this, Reggiani et al. (2002) demonstrated the applicability of resilience analysis to economic systems, particularly in spatial economics [20]. Thereafter, a surge of research on economic resilience has emerged and the concept of regional economic resilience has become increasingly sophisticated. Simmie and Martin (2010) synthesized prior theories to construct an analytical framework for economic resilience, positing that resilience determines regions’ differential capacities to withstand shocks, and that economic resilience research should prioritize analyzing the dynamic evolutionary process of system adaptive capacities. They proposed that the concept of regional economic resilience must, at a minimum, encompass three core capabilities: resistance to external shocks, the capacity to maintain operational continuity through resource reallocation following the shock, and the capacity to re-structural through adaptive transformation [21]. Building on this foundation, Martin (2012) clarified the concepts related to shocks, resilience, and post-shock trajectories. He argued that a region’s economic growth rate and structural characteristics determine its capacity to withstand shocks. The development trajectories of a region’s enterprises and industries shape its ability to adapt to shocks, while shock characteristics can alter the region’s post-crisis economic development path. Consequently, regional economic resilience must encompass multiple layers of meaning, including resistance, recovery, re-orientation, and renewal [22].

Martin and Gardiner (2019) discovered that the performance and influencing factors of urban economic resilience vary across different shocks, because economic resilience is not static but evolves dynamically over time [23]. Therefore, it’s necessary to employ a more granular set of urban indicators to accurately measure urban economic resilience and assess the impact of the pandemic. The selection of our metrics for measuring urban economic resilience is primarily based on the concept of economic resilience as developed in the work of Simmie and Martin (2010) [21] and Martin (2012) [22]. Three dimensions of capability are considered: First, the capacity for shock resistance determines whether an urban economic system fractures under external pressure or instead manages to absorb disturbances with minimal fluctuation. Second, the capacity for adaptive recovery depends on the resources available for restoring operational order and determines the extent to which pre-shock functions, such as routine economic activities, can be restored after the shock. Third, the capacity for innovative transformation reflects the system’s capacity for post-shock learning and enables the development of new economic forms or the restructuring of existing activities.

Several methods exist for measuring economic resilience. The first method uses economic sensitivity indicators to assess resilience. Martin (2012) used output and employment as proxies to examine regional economic resilience across three recessions in the UK from 1979 to 2010, finding that employment took longer to recover than output [22]. Brakman et al. (2015) adopted this approach, using the 2008 economic and financial crisis as the shock event, and calculated the resilience sensitivity indicators of 207 European regions from 22 countries [24]. Due to data limitations covering only 2008–2012 period, their study focused on assessing regional capacities during the initial shock or resistance phase. While the sensitivity indicator approach streamlines analysis and enhances operational feasibility, its reliance on one or two metrics (primarily employment and output) can lead to an oversimplification of economic resilience, failing to fully capture the multidimensional responses of urban systems to the shock. The second method involves constructing functional models to estimate resilience, an approach frequently adopted in studies analyzing natural disasters as exogenous shocks. The idea is to develop econometric models that simulate hypothetical growth trajectories for economic systems, with resilience measured by the gap between actual post-shock performance and counterfactual growth projections. A smaller divergence indicates less systemic damage and higher economic resilience. The approach has been applied in the following empirical studies. Zhou et al. (2019) analyzed panel data from ten counties affected by Wenchuan earthquake to compute regional resilience indices [25]. Luo et al. (2024) calculated the economic resilience indices for five earthquake- stricken counties and four adjacent counties in Sichuan Province following the 2017 M7.0 Jiuzhaigou earthquake, it revealed that disaster-stricken areas would recover to pre-earthquake levels within two years [26]. This method depends critically on accurately predicting the post-disaster economic growth path. As a result, it is best suited for quantifying resilience in the period immediately following a shock and in the epicenter regions, but is not a versatile tool for arbitrary time frames and locations. The third method involves constructing a comprehensive indicator system. Li et al. (2021) developed a framework comprising three dimensions——pressure resistance, growth momentum, and potential development——to assess economic resilience in Liaoning Province. The analysis concluded that capacity of pressure resistance is pivotal in shaping the resilience trajectory of such old industrial bases [27]. Cai et al. (2024) adopted a four-dimensional system comprising risk, stability, circulation, and innovation indices to evaluate economic resilience of 31 Chinese provinces from 2008 to 2019, and revealed expanding absolute and relative disparities between the northern and southern regions [28]. Although this multidimensional approach provides benefits like broad indicator coverage, diversified measurement perspectives, and systematic representation of complex economic systems, it carries the inherent risk of subjectivity in the selection of indicators. Given the multifaceted nature of economic resilience and its dynamic evolutionary characteristics, we contend that constructing a comprehensive indicator system is the most appropriate methodological approach for this study. In constructing the indicator system and selecting measurement methods, we will take measures to minimize the influence of subjectivity.

### 2.2 The direct impact of COVID-19 on urban economic resilience

A healthy population is a fundamental prerequisite for stable economic growth and urban economic resilience, for at least four reasons. First, it ensures a productive labor supply, raises household incomes, and enhances overall urban output. Second, improved life expectancy and reduced morbidity accelerate human capital accumulation. Third, it enables long-term planning for individuals and households, leading to higher social savings and investments. Fourth, lower child mortality contributes to reduced fertility rates and promotes female labor force participation [29]. Public health emergencies like COVID-19, by directly impairing population health, undermine urban economic resilience through the channels outlined above, especially in contexts lacking effective disease prevention or treatment measures [5]. Moreover, government measures to contain COVID-19 impede the recovery of urban economic by increasing public fiscal expenditures and raising general economic operational costs. Several studies have indicated that COVID-19 containment policies imposed significant economic costs. One study estimated that a 30-day containment policy would reduce industrial output by approximately 10%, noting that measures such as workplaces closures and international travel restrictions were also highly costly [30]. COVID-19 containment policies restricted the flow of labor and production materials, which hindered business resumption and disrupted global supply chain operations. Furthermore, the rapid spread of the pandemic caused breakdowns at multiple points within global industrial chains [31]. Governments, thus, would have less tax revenues from households and enterprises [32]. Garud and Karnøe (2001) put up an interesting idea that organizations can deliberately lock out from established path dependencies through conscious actions, particularly by implementing “intended deviation” from existing trajectories [32]. This concept of intended deviation emphasizes the agentic capacity of various entities in the urban economic system, which may be activated by exogenous shocks [33]. As an external disruptive event, COVID-19 broke the urban’s reliance on legacy technological regimes, accelerating a shift toward emerging technological systems. The pandemic compelled entities in the city to adopt more productive innovations [34]. We therefore deem that COVID-19 adversely affects urban economic resilience, primarily impairing the sub-dimensions of shock resistance and adaptive recovery, while its impact on innovative transformation dimension remains ambiguous.

### 2.3 The transmission channels of COVID-19’s impact on urban economic resilience

Regarding supply-side channels, COVID-19-induced health shocks impaired the labor supply by affecting workers’ physiological and psychological well-being. At the physiological level, a large number of infected people developed Long COVID, which can prevent them from working and lead to labor supply shortages [35]. According to the research from Brookings Institution, if individuals with Long COVID reduced their working hours by a quarter, the equivalent loss in full-time employment would represent approximately 1.6 million workers, explaining roughly 15% of job vacancies in the U.S. during January 2022 [36]. Labor supply is further constrained by absenteeism among healthy workers, driven by concern about infection. This challenge was especially pronounced in high-contact sectors such as healthcare and personal services, where wages may be inadequate to offset the associated health risks [37]. At the psychological level, COVID-19 elevated mental health disorders and altered risk perceptions across global populations. Following the pandemic, the global prevalence of major depressive disorders and anxiety disorders has increased by 27.6% and 25.6%, respectively [38]. Individual tracking surveys in China indicated that COVID-19 containment measures delayed work resumption, leading to job losses and re-employment difficulties. Both unemployment anxiety and fear of infection notably impaired short-term psychological well-being [39]. From the corporate perspective, the outbreak of COVID-19 coincided with China’s Spring Festival, which not only severely disrupted the post-holiday return of the workforce but also exacerbated structural contradictions in the labor market, thereby adversely impacting enterprise production and operations [40]. Amid the pandemic shock, business risks and uncertainties surged abruptly, while most Chinese small and micro enterprises faced heightened financial fragility. In the early stages of the pandemic, 85% of enterprises held cash reserves insufficient to sustain operations for three months. Even government rescue policies proved inadequate to alleviate these urgent liquidity pressures in the short term, which meant many enterprises were unable to survive [41]. Surviving enterprises were compelled to implement cost-cutting strategies. These strategies included workforce reductions and salary cuts to mitigate risk, as well as accelerated adoption of new technologies to reduce labor dependence and minimize contact-intensive operations. The resultant substitution effects accelerated structural adjustments in the labor market [9]. Consequently, COVID-19 depressed aggregate employment levels and altered the employment structure, constraining the recovery of economic system.

Regarding the demand side, research has focused on consumption and investment channels. The impact of COVID-19 on the consumption channel can be categorized into five main aspects. First, elevated morbidity and mortality rates diminish aggregate urban consumption. Second, individuals reduced spending in public settings to avoid exposure. Third, health concerns and general uncertainty prompt a shift from consumption to increased precautionary savings. Fourth, income loss due to illness or unemployment directly reduces disposable income and spending. Fifth, consumption structures shifted toward expenditures targeting disease prevention or treatment [29]. The analytics firm Earnest Research assessed COVID-19’s impact on consumption by tracking credit and debit card spending of nearly 6 million Americans. Its data for the week beginning April 1, 2020, found that spending on airlines, hotels, car rentals, taxis, ridesharing, and cinemas declined by 75%−95% compared to the same period in 2019 [42]. Additionally, studies on the experience effect suggest that the human brain is highly sensitive to experiences, and the feelings arising from these events are imprinted as memory anchors which then become internalized as beliefs. Major adverse events shape long-term consumption tendencies, affecting not only immediate spending but also future expectations, thereby reducing consumption levels [43]. In summary, COVID-19 not only impacted consumption in the short term but also triggered prolonged sluggishness in consumer demand. Changes in consumption demand induce shifts in industrial spatial layout and market scale, thereby affecting urban economic resilience [44]. Collectively, COVID-19 adversely impacts urban economic resilience through consumption channels. The impact of COVID-19 on the investment channel manifests in two primary forms: tangible capital investment and intangible capital investment. First, several factors may collectively suppress capital investment for months or even longer: cash-flow constraints stemming from containment measures, uncertainty about the pandemic’s trajectory, public concerns over short-to-medium-term income reductions, and uncertainty regarding economic growth prospects and product demand. These factors inevitably reduced productive capital stock across economies. Furthermore, pandemic-induced shifts in the structure of demand and persistent health concerns have devalued specific asset classes, such as large entertainment venues, high-density retail facilities, and high-volume restaurants. Second, R&D investment is highly vulnerable to economic uncertainty. Given its irreversible nature and higher risk profile compared to tangible capital investment, uncertainty shocks inflict disproportionate damage on this innovation-oriented capital [45]. The exceptionally high economic uncertainty during the pandemic [46] likely suppressed investment rates in R&D-intensive intangible capital, at least temporarily. As a key component of aggregate demand, such investment directly determines short-term output. In the long term, it provides the material and technological foundation for production, thereby fostering economic growth [47]. Thus, COVID-19 induces investment stagnation, thereby adversely affecting urban economic resilience.

Regarding industrial channels, industrial structure is a significant determinant of urban economic resilience [11]. Academic debates focus intensely on industrial specialization agglomeration and diversification agglomeration. The core logic of industrial specialization rests on Marshallian externalities [48], which emphasize that the geographic concentration of firms in similar industries facilitates access to specialized information and accelerates the diffusion of technology. The main idea of industrial diversification relies on Jacobs externalities, which stress the economic linkages, knowledge spillovers, and technological complementarities that occur across diverse industries [49]. The resulting innovation activities are crucial for renewal of economic growth paths and recombination of resources. Studies of major shocks, such as 2008 financial crisis, have revealed that diversified industrial agglomeration functions as an economic stabilizer by cushioning volatility during severe external shocks [50]. Further research distinguishes between related diversification and unrelated diversification, indicating that the risk dispersion effect in the early stages of a financial crisis mainly originates from the latter. Accordingly, cities with higher levels of unrelated diversification were better cushioned, as this form of diversification significantly bolstered economic resilience by fostering new economic innovations [51]. Because the COVID-19 shock was fundamentally different from the 2008 financial crisis, the manifestation of urban economic resilience and its key determinants are likely to differ considerably [52]. COVID-19 impacted all industries comprehensively. In the early phase of the pandemic, few industrial sectors were immune to shutdowns, with both the secondary and tertiary industries being severely affected [11]. In face of such a widespread shock, overly diversified structures may be less effective in dispersing impacts, potentially weakening regional resistance [17]. Does industrial diversification continue to function as an economic stabilizer, or has it become a channel transmission for shocks such as COVID-19? This warrants further exploration. Therefore, we hypothesize:

H1: COVID-19 adversely affected urban economic resilience. At the sub-dimensional level, it exerted negative impacts on the sub-dimensions of shock resistance and adaptive recovery, while a positive impact on the innovative transformation dimension.

H2: COVID-19 impacts urban economic resilience through several transmission channels, including aggregate employment, employment structure, consumption, investment, and industrial diversification.

**Fig 2:**
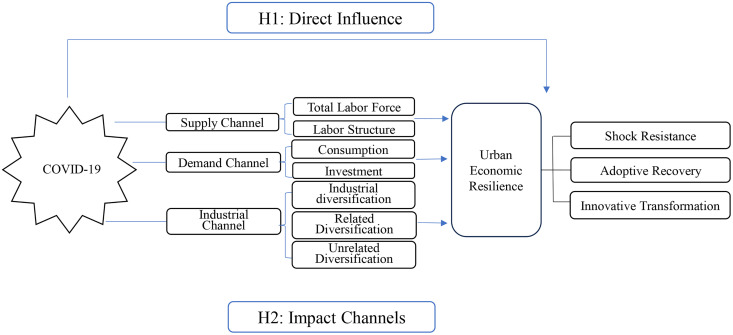
Theoretical model.

To illustrate our hypothesis, we present Fig 2.

## 3 Methodology

### 3.1 Samples and data sources

To ensure urban data standardization and account for the phased conclusion of COVID-19 in China by December 2022, our study focuses on the period from 2017 to 2022. After excluding cities with substantial data gaps, our final sample comprises 281 prefecture-level and above cities with relatively complete data. The data were primarily sourced from provincial and municipal statistical yearbooks, the China City Statistical Yearbook, the WIND database, and the CSMAR database. All data involving prices were adjusted for price changes. For partially missing data, the imputation method was applied for supplementation.

### 3.2 Variables and measurement

#### 3.2.1 Dependent variable.

The dependent variable in this study is urban economic resilience (denoted by *res*). Numerous researchers have contributed to the conceptualization of urban economic resilience [21,22]). Although terminology varies, these studies consistently encompass three capabilities of an economy: the ability to maintain economic functioning, mobilize resources for recovery, and capacity for reconstruction and transformation. Based on these concepts, this study contends that urban economic resilience should be measured through comprehensive indicators across three sub-dimensions——shock resistance, adaptive recovery, and innovative transformation——which correspond respectively to the three capabilities in the economic resilience concept. The shock resistance dimension determines an economy’s vulnerability to external shocks; the adaptive recovery dimension determines the extent to which an urban economy can recover to its pre-shock level after an impact; and the innovative transformation dimension determines the ability to achieve structural transformation through post-shock adjustment and innovation. Building upon studies of Gong et al. (2022) [53] and Zhang and Dai (2023) [54], we measure these three sub-dimensions by constructing a comprehensive indicator system for evaluating urban economic resilience. The indicator system is shown in Table 1, where we specify the measurement metrics, attributes, and literature references for all 21 indicators.

**Table 1:** Urban economic resilience indicator system.

	Sub-dimension	Indicators	Indicators Metrics	Indicator attributes	References
Urban economic resilience, *res*	Shock resistance, *r*_*1*_	Economic foundation	City-level GDP/ National-level GDP, reflecting the relative fluctuations of the urban economy	Positive	Zhang et al. (2018) [55]; Gong et al. (2022) [53]
		Economic growth (%)	GDP grow rate, reflecting the absolute fluctuations of the urban economy	Positive	Cui (2021) [56]; Gao et al. (2022) [57]; Tang et al. (2022) [58]
		Loan-to-deposit ratio	Loan-to-deposit ratio of financial institutions, reflecting liquidity risk	Negative	Gong et al. (2022) [53]; Zhang and Yao (2023) [59]
		Registered urban unemployment rate (%)	Reflecting the fluctuations in the labor market	Negative	Zhu and Sun (2021) [60]; Zhang and Yao (2023) [59]; Hao and Yan (2023) [61]
		Per capita disposable income of urban residents (Yuan)	Reflecting the family’s income situation and consumption potential	Positive	Gao et al. (2022) [57]; Hao and Yan (2023) [61]; Shi et al. (2023) [62]
		Dependence of foreign trade	Import and export amounts, the degree of the economy of a city relied on foreign trade	Negative	Gao et al. (2022) [57]; Gong et al. (2022) [53]; Zhang and Yao (2023) [59]; Zhang (2025) [63]
		Unemployment insurance rate (%)	Number of employees participating in unemployment insurance/Urban population (%), reflecting the employment security situation of the city	Positive	Gao et al. (2022) [57]; Gong et al. (2022) [53]; Zhang (2025) [63]
		Medical insurance rate (%)	Number of employees participating in medical insurance/Urban population, reflecting the medical security situation of the city	Positive	Gao et al. (2022) [57]; Shi et al. (2023) [62]
	Adaptive recovery, *r*_*2*_	Growth rate of investment in fixed assets (%)	The extent of the recovery in investor confidence	Positive	Zhu and Sun (2021) [60]; Gong et al. (2022) [53]
		Growth rate of total retail sales of consumer goods (%)	The level of economic activity and the recovery of consumer confidence	Positive	Zhu and Sun (2021) [60]; Gao et al. (2022) [57]; Gong et al. (2022) [53]; Shi et al. (2023) [62]
		Fiscal balance rate (%)	Revenues/ Expense from the general budget of the local government (%), reflecting financial self-sufficiency and financial health status of a city	Positive	Zhu and Sun (2021) [60]; Gao et al. (2022) [57]; Gong et al. (2022) [53]; Zhang and Yao (2023) [59]
		Loan-to-GDP ratio (%)	Total loan/ GDP, reflecting the credit demand and the recovery situation of a city	Positive	Zhu and Sun (2021) [60]; Gao et al. (2022) [57]
		Income gap between urban and rural residents	Per capita disposable income of urban residents/ Per capita disposable income of rural residents, reflecting the fairness of social resource distribution	Negative	Zhu and Sun (2021) [60]; Shi et al. (2023) [62]
		Highway passenger transport volume per capita	Highway passenger volume/ population, reflecting the situation of personnel mobility	Positive	Zhang et al. (2018) [55]; Pan and Tian (2023) [64]
		Highway cargo transport volume per capita	Highway cargo volume/ population, reflecting the operational status of freight and supply chain	Positive	Lu et al. (2020) [65]; Fan and Lu (2020) [66]
	Innovative transformation, *r*_*3*_	Industrial upgrading	Added value of tertiary industry/ City-level GDP, Reflecting the industrial advancement level	Positive	Zhu and Sun (2021) [60]; Gao et al. (2022) [57]; Zhang and Yao (2023) [59]; Hao and Yan (2023) [61]
		Regional educational focus	Education expenses of local government/ Total Expense of local government. The prioritization level of education by local governments	Positive	Zhu and Sun (2021) [60]; Hao and Yan (2023) [61]; Zhang (2025) [63]
		Human capital potential	Number of college students per 10,000 people, reflecting the penetration level of higher education	Positive	Gong et al. (2022) [53]; Shi et al. (2023) [62]; Zhang and Yao (2023) [59]; Hao and Yan (2023) [61]
		Urbanization rate (%)	The degree of concentration of population from rural to urban	Positive	Zhu and Sun (2021) [60]; Gao et al. (2022) [57]; Zhang and Dai (2023) [54]
		Urban sewage discharge (10,000 cubic meters)	Reflecting the degree of water pollution.	Negative	Lu et al. (2020) [65]; Gao et al. (2022) [57]; Shi et al. (2023) [62]; Zhang and Dai (2023) [54]
		Carbon dioxide emission (10,000 tons)	Reflecting the degree of air pollution.	Negative	Zheng et al. (2013) [67]; Liu et al. (2021) [68]

Following the construction of urban economic resilience indicator system, we employed the entropy method to calculate its comprehensive score across China. Rooted in the principle of entropy from physics, this method first identifies the degree of dispersion of each indicator. It then computes the information entropy of the data to assign corresponding weights to the underlying indicators, ultimately synthesizing them into a comprehensive evaluation score. The specific computational procedure proceeds as follows:

Step one: Each indicator is standardized. We designate *x*_*tnm*_ as the value of the indicator *m* for city *n* in year *t*, where *m,n,t* (*m = 1,2,…,M; n = 1,2,…,N; t = 1,2,…,T*). The calculation of standardization for positive variables is: $X_{tnm}=[x_{tnm}-min(x_{tnm})]/[{max(x}_{tnm})-min(x_{tnm})]$. The calculation of standardization for negative variables is: $X_{tnm}=[{max(x}_{tnm})-x_{tnm}]/[{max(x}_{tnm})-min(x_{tnm})]$.

Step two: This step objectively determines indicator weights based on the inherent dispersion (information entropy) of the data, thereby calculating a comprehensive score of urban economic resilience. Firstly, we calculated value of *m*th indicator of the *n*th city as a proportion of overall indicators: $P_{tnm}=X_{tnm}/\sum_{t}^{T}\sum_{n}^{N}X_{tnm}$. Secondly, entropy value of each indicator is calculated as follows: $E_{m}=-k\sum_{t}^{T}\sum_{n}^{N}P_{tnm}\text{ln}(P_{tnm})$, in which k=1/ln(T*N). Thirdly, compute the information utility value: $D_{m}=\text{1}-E_{m}$. Then, we calculated the weight of *m*th indicator: $W_{m}=D_{m}/\sum_{m}^{M}D_{m}$. Lastly, the comprehensive score of urban economic resilience is derived: ${Score}_{tn}=\sum_{m}^{M}(W_{m}*X_{tnm})$.

#### 3.2.2 Independent variable.

The independent variable is the severity of COVID-19 pandemic (denoted by *cov*). Following An and Huang (2024) [10], we compiled daily confirmed COVID-19 cases for sample cities to measure the pandemic’s severity and aggregated them into annual figures for regression analysis. This indicator is expressed in ten thousand cases.

#### 3.2.3 Mediating variables.

(1) Aggregate employment is proxied by the surveyed urban unemployment rate [69], denoted by *unemp*.
(2) Employment structure is measured by employment of sectoral employment shares [70]. Manufacturing employment is measured by the ratio of urban manufacturing employment to total employment, denoted by *manu*. Service sector employment is measured by the ratio of urban service employment to total employment, denoted by *serv*. The service sector is further subdivided into producer services and high-end services. The former is measured by the ratio of urban employment in producer services to total service employment, denoted by *ps*. The latter is measured by the ratio of urban employment in high-end services to total service employment, denoted by *hes*.
(3) Consumption is proxied by per capita total retail sales of consumer goods [71], denoted by *cons*.
(4) Investment is proxied by the investment growth rate [72], denoted by *inv*.
(5) The industrial diversification (denoted by *div*) is measured by calculating the diversification entropy index [51]: $div=\sum_{h=\text{1}}^{m}l_{h}/ln(\text{1}/l_{h})$. Diversification can be decomposed into unrelated diversification (denoted by *uv*) and related diversification (denoted by *rv*): $uv=\sum_{g=\text{1}}^{G}l_{g}ln(\frac{\text{1}}{l_{g}})$, $rv=\sum_{g=\text{1}}^{G}l_{g}h_{g}$, *in which* $h_{g}=\sum_{h\in g}{(l_{h}/l}_{g})ln{(l_{h}/l}_{g})$. $l_{g}=\sum_{h\in g}l_{h}$, $l_{g}$ denotes employment share of industry sector *g*. $l_{h}$ denotes employment share of industry sub-sector *h* (*h* = *1, 2, …, m*). *m* denotes 19 industries classified by the China City Statistical Yearbook.

#### 3.2.4 Control variables.

This study includes seven control variables may influence urban economic resilience [17,51,70]: (1) Trade openness (*open*) is measured by the ratio of total imports and exports to urban GDP. (2) Industrial structure (*ind*) is measured by the ratio of the value added from secondary sector to that from tertiary sector. (3) Financial development level (*fin*) is quantified as the ratio of year-end financial institution loan balances to urban GDP. (4) Population agglomeration (*pop*) is measured using urban population per unit area. (5) Informatization level (*inf*) is proxied by internet penetration rate. (6) The level of foreign direct investment (*fdi*) is measured by the ratio of utilized foreign direct investment to urban GDP. (7) Urbanization level (*urban*) is measured by the proportion of urban population to total population.

### 3.3 Fixed-effect models

#### 3.3.1 Baseline regression model.

This study employs a two-way fixed effects model to examine the impact of COVID-19 on urban economic resilience. The model includes individual fixed effects to address time-invariant omitted variable bias across units and time fixed effects to account for common shocks over time. It enables a more credible identification of the independent variable’s effect, substantially enhancing the reliability of causal inference and mitigating certain sources of endogeneity. The model is set as shown in Eq (1):


$${\;Y}_{i,t}=\alpha_{\text{0}}+\alpha_{\text{1}}{cov}_{i,t}+\alpha_{c}{controls}_{i,t}+\mu_{i}+\delta_{t}+\epsilon_{\text{1},it}\;\;$$ (1)

In Eq (1), *Y*_*i,t*_ denotes the level of urban economic resilience and sub-dimensions for city *i* in year *t*; *cov*_*i,t*_ measures pandemic severity for city *i* in year *t*; *controls*_*i,t*_ represents control variable; *μ*_*i*_ is an individual fixed effect; *δ*_*t*_ is a time fixed effect; *ε*_*i,*t_ is a random disturbance term; and *α* is the parameter to be estimated.

#### 3.3.2 Intermediary effect model.

To examine the transmission channels through which COVID-19 affects urban economic resilience, we specify the mediating effect models based on Baron and Kenny (1986) [73] and Wen et al (2004) [74]. The models are set as shown in Eq (2) and (3):


$$\;\;M_{i,t}=\beta_{\text{0}}+\beta_{\text{1}}{cov}_{i,t}+\beta_{c}{controls}_{i,t}+\mu_{i}+\delta_{t}+\epsilon_{\text{2},it}$$ (2)

$${\;Y}_{i,t}=\gamma_{\text{0}}+\gamma_{\text{1}}{cov}_{i,t}+\gamma_{\text{2}}M_{i,t}+\gamma_{c}{controls}_{i,t}+\mu_{i}+\delta_{t}+\epsilon_{\text{3},it}\;\;$$ (3)

Where *M*_*i,t*_ is the mediating variable, representing different transmission channels; *ε*_2,*it*_, *ε*_3,*it*_ are random disturbance terms; and *β*, *γ* are parameters to be estimated. The transmission channels posited in Hypothesis 2 are tested by jointly examining Eq (1) to (3). The significant coefficient *α*_*1*_ in Eq (1) provides initial support for Hypothesis 1, indicating a total effect. The analysis then proceeds to Eq (2) and Eq (3). A significant coefficient *β*_*1*_ suggests the presence of a mediating effect. Finally, if the coefficient *γ*_*1*_ in Eq (3) is either insignificant or remains significant but its absolute value is less than that of *α*_*1*_, it can be concluded that the hypothesized transmission channels mediate the impact of COVID-19 on urban economic resilience.

## 4 Empirical results

### 4.1 Descriptive statistics

Table 2 presents descriptive statistics for all variables. Urban economic resilience ranges from 0.0635 to 0.5251 with a mean of 0.1667 and a standard deviation of 0.083, indicating substantial variations across the 281 sample cities. Confirmed COVID-19 cases varied widely across cities, from 0 to 63,042. Among the mediating variables, consumption exhibited the largest standard deviation (1.2479), highlighting substantial disparities in consumption levels.

**Table 2:** Descriptive statistics for the main variables.

Variables	Sign	Obs	Mean	SD	Min	Max
Dependent variables	res	1686	0.1667	0.0830	0.0635	0.5251
	*r*_*1*_	1686	0.0591	0.0428	0.0135	0.3299
	*r*_*2*_	1686	0.0498	0.0188	0.0180	0.2491
	*r*_*3*_	1686	0.0577	0.0363	0.0176	0.2276
Independent variable	cov	1686	0.0225	0.2492	0.0000	6.3042
Mediating variables	unemp	1686	2.9245	0.7862	0.3100	6.1200
	manu	1686	0.2079	0.1227	0.0092	0.7803
	serv	1686	0.5888	0.1324	0.1657	0.9418
	ps	1686	0.2335	0.0805	0.0797	0.5474
	hes	1686	0.1761	0.0674	0.0356	0.8009
	cons	1686	2.3810	1.2479	0.0840	7.9524
	inv	1686	0.0654	0.1105	−0.5970	0.4171
	div	1686	2.2639	0.2085	1.0899	2.7206
	uv	1686	0.6816	0.0912	0.2438	1.0979
	rv	1686	1.5824	0.2218	0.5906	2.2673
Control variables	open	1686	0.1712	0.2563	0.0004	2.4913
	ind	1686	0.9109	0.3688	0.1770	3.1124
	pop	1686	0.3206	0.2091	0.0370	2.3236
	fin	1686	1.2480	0.6890	0.0001	10.2438
	inf	1686	0.3475	0.1768	0.0573	1.4857
	fdi	1686	0.0241	0.0835	0.0000	1.1977
	urban	1686	0.6049	0.1343	0.2503	1.0000

### 4.2 Baseline results

Table 3 presents the baseline regression results for the impact of COVID-19 on urban economic resilience and its sub-dimensions. Columns (1)-(4) show effects without control variables. Specifically, Column (1) shows the impact of COVID-19 on overall urban economic resilience, while Columns (2)-(4) show the impacts on sub-dimensions of shock resistance, adaptive recovery, and innovative transformation. Columns (5)-(8) display the results with control variables. The sign and statistical significance of *cov* remain consistent with and without control variables. Column (5) indicates that every additional 10,000 confirmed COVID-19 cases reduce urban economic resilience by 0.0020 units, confirming an adverse pandemic effect. Results in Columns (6) and (7) show that COVID-19 adversely affected the shock resistance and adaptive recovery dimensions, with the effect on the latter being significant at the 1% level. Although the shock resistance coefficient is insignificant, its negative sign partially supports our theoretical hypothesis. We attribute the lack of a statistically significant effect of COVID-19 on the shock resistance dimension to two factors: the recurrent and spatially uneven nature of the pandemic’s spread, and the differential resistance responses arising from urban heterogeneity (e.g., city scale, location). These findings align with the research of An and Huang (2024) [10]. Column (8) shows a positive but statistically insignificant effect of COVID-19 on the innovative transformation dimension. This finding resonates with conclusions from research on other public health shocks. For instance, scholars have argued that labor scarcity following the Black Death spurred institutional innovations in guild systems and the development of labor-saving technologies [75]. Similarly, COVID-19 has accelerated technological progress in medical-related fields and compelled widespread innovations in digital technologies globally. However, given the significant short-term negative effect of COVID-19 on technological innovation activities [34], we posit that its positive impact on the dimension of innovative transformation may be subject to a time lag. This lag effect could explain the statistically insignificant results in Columns (4) and (8).

**Table 3:** Baseline regression results of the impact of COVID-19 on urban economic resilience.

Variables	*res* (1)	*r*_*1*_ (2)	*r*_*2*_ (3)	*r*_*3*_ (4)	*res* (5)	*r*_*1*_ (6)	*r*_*2*_ (7)	*r*_*3*_ (8)
cov	-0.0017^**^ (0.0008) [-0.003,0.000]	−0.0007 (0.0006) [-0.002,0.000]	−0.0017^***^ (0.0005) [-0.003, -0.001]	0.0007 (0.0008) [-0.001,0.002]	−0.0020^**^ (0.0009) [-0.004, -0.000]	−0.0008 (0.0005) [-0.002, 0.000]	−0.0019^***^ (0.0007) [-0.003, -0.001]	0.0007 (0.0008) [-0.001, 0.002]
open					0.0175^**^ (0.0069) [0.004, 0.031]	0.0017 (0.0035) [-0.005, 0.009]	0.0034 (0.0024) [-0.001, 0.008]	0.0124^**^ (0.0053) [0.002, 0.023]
ind					-0.0030 (0.0024) [-0.008, 0.002]	0.0035^***^ (0.0008) [0.002, 0.005]	−0.0004 (0.0021) [-0.005, 0.004]	−0.0062^***^ (0.0010) [-0.008, -0.004]
pop					-0.0050 (0.0035) [-0.012, 0.002]	−0.0009 (0.0018) [-0.004, 0.003]	−0.0013 (0.0016) [-0.004, 0.002]	−0.0028 (0.0022) [-0.007, 0.001]
fin					0.0037^***^ (0.0006) [0.003, 0.005]	−0.0007^*^ (0.0004) [-0.002, 0.000]	0.0038^***^ (0.0003) [0.003, 0.005]	0.0006^*^ (0.0003) [-0.000, 0.001]
inf					0.0105 (0.0074) [-0.004, 0.025]	0.0019 (0.0033) [-0.005, 0.009]	−0.0017 (0.0047) [-0.011, 0.008]	0.0103^**^ (0.0043) [0.002, 0.019]
fdi					-0.0262^***^ (0.0080) [-0.042, -0.010]	−0.0178^***^ (0.0034) [-0.025, -0.011]	0.0021 (0.0043) [-0.006, 0.011]	−0.0104^**^ (0.0052) [-0.021, -0.000]
urban					0.0032 (0.0116) [-0.020, 0.026]	−0.0394^***^ (0.0066) [-0.052, -0.027]	0.0068 (0.0057) [-0.004, 0.018]	0.0358^***^ (0.0042) [0.028, 0.044]
City fixed effect	YES	YES	YES	YES	YES	YES	YES	YES
Year fixed effect	YES	YES	YES	YES	YES	YES	YES	YES
Observations	1686	1686	1686	1686	1686	1686	1686	1686
R^2^	0.9930	0.9929	0.9388	0.9875	0.9934	0.9938	0.9436	0.9895

Note: *, **, *** denote significance at the 10%, 5%, and 1% levels, respectively. The values in parentheses are cluster-robust standard errors at the city level. The values in bracket are 95% confidence intervals.

### 4.3 Endogeneity problem treatment

This study employs Propensity Score Matching (PSM) approach and Difference-in-Differences (DID) models to mitigate endogeneity in baseline regression. To mitigate endogeneity from sample selection, we employ the PSM approach following Zhang et al. (2021) [76]. Cities are classified into treatment and control groups based on a threshold of pandemic severity. Given the right-skewed distribution of confirmed COVID-19 cases, the mean is an upward-biased statistic. Therefore, we used the two-thirds quantile (ascending order) of confirmed cases as the cutoff point. Cities with values above this threshold were assigned to the treatment group, and those below it to the control group. Using urban characteristics as covariates, we performed 1:1 nearest-neighbor matching. The regression was then re-estimated on the matched sample, and the corresponding results are presented in Table 4. Columns (1)-(4) display results without control variables, Columns (5)-(8) display results with control variables. The regression results indicate that COVID-19 exerted significant negative impacts on overall urban economic resilience and on the sub-dimensions of shock resistance and adaptive recovery, while its effect on the innovative transformation dimension was positive but statistically insignificant. These results are largely consistent with the baseline regression results.

**Table 4:** Results of endogeneity treatment: PSM.

Variables	res (1)	*r*_*1*_ (2)	*r*_*2*_ (3)	*r*_*3*_ (4)	res (5)	*r*_*1*_ (6)	*r*_*2*_ (7)	*r*_*3*_ (8)
cov	-0.0062^***^ (0.0020)	−0.0039^***^ (0.0012)	−0.0028^*^ (0.0017)	0.0006 (0.0010)	−0.0069^***^ (0.0019)	−0.0036^***^ (0.0010)	−0.0034^**^ (0.0015)	0.0001 (0.0011)
Control Variables	NO	NO	NO	NO	YES	YES	YES	YES
City fixed effect	YES	YES	YES	YES	YES	YES	YES	YES
Year fixed effect	YES	YES	YES	YES	YES	YES	YES	YES
Observations	731	731	731	731	731	731	731	731
R^2^	0.9897	0.9865	0.9402	0.9888	0.9903	0.9877	0.9468	0.9905

Note: *, **, *** denote significance at the 10%, 5%, and 1% levels, respectively. The values in parentheses are cluster-robust standard errors at the city level.

Furthermore, following the methodology of Yi and Zhao (2024) [77], we employ a Difference-in-Differences (DID) model and construct the *Treat* and *Post* variables. Adopting the uniform approach to periodization established by Nunn and Qian (2011) [78], we define the *Post* dummy variable as followings: years from 2020 onward are coded as 1, and years before 2020 are coded as 0. For city classification, based on the research of Liu et al. (2024) [79], sample cities are classified into epicenter (*Treat* = 1) and non-epicenter (*Treat* = 0) groups according to the number of confirmed COVID-19 cases. Shanghai, Wuhan, Guangzhou, Beijing, and 90 other cities with confirmed case counts above the two-thirds quantile threshold are classified as epicenters. These cities collectively account for 95% of all confirmed COVID-19 cases in China. Cities with confirmed case counts below the two-thirds quantile threshold are classified as non-epicenters. Table 5 presents the regression results using *Treat×Post* as the independent variable. Columns (1)-(4) report results without control variables, and Columns (5)-(8) display results with control variables. Results in Column (5) indicates that *Treat×Post* negatively affects overall urban economic resilience. At the sub-dimension level, the results in Columns (6)-(8) reveal that *Treat×Post* exerts significant negative impacts on the sub-dimensions of shock resistance and adaptive recovery, but a significant positive impact on the innovative transformation dimension. Therefore, when treating COVID-19 as a natural experiment, the baseline findings regarding the impact of the pandemic on overall urban economic resilience and its sub-dimensions remain robust.

**Table 5:** Results of endogeneity treatment: DID.

Variables	res (1)	*r*_*1*_ (2)	*r*_*2*_ (3)	*r*_*3*_ (4)	res (5)	*r*_*1*_ (6)	*r*_*2*_ (7)	*r*_*3*_ (8)
Treat×Post	-0.0015 (0.0015)	−0.0014^*^ (0.0007)	−0.0012 (0.0009)	0.0011 (0.0009)	−0.0015 (0.0014)	−0.0016^**^ (0.0006)	−0.0015^*^ (0.0009)	0.0016^*^ (0.0008)
Control Variables	NO	NO	NO	NO	YES	YES	YES	YES
City fixed effect	YES	YES	YES	YES	YES	YES	YES	YES
Year fixed effect	YES	YES	YES	YES	YES	YES	YES	YES
Observations	1686	1686	1686	1686	1686	1686	1686	1686
R^2^	0.9930	0.9929	0.9386	0.9875	0.9934	0.9939	0.9434	0.9896

Note: *, **, *** denote significance at the 10%, 5%, and 1% levels, respectively. The values in parentheses are cluster-robust standard errors at the city level.

### 4.4 Transmission channels analysis

Using Eq (1)-(3), we examine the transmission channels through which COVID-19 affects urban economic resilience. Table 6 reports the effects of the pandemic on urban economic resilience through the transmission channels of aggregate employment and employment structure, while Table 7 shows the effects through the channels of consumption, investment, and industrial diversification.

**Table 6:** Regression results: transmission channels analysis I.

Variables	res (1)	Aggregate Employment		Employment Structure							
				Manufacturing employment		Service employment		Producer services employment		High-end services employment	
		unemp (2)	res (3)	manu (4)	res (5)	serv (6)	res (7)	ps (8)	res (9)	hes (10)	res (11)
cov	-0.0020** (0.0009)	−0.03912 (0.1057)	/	−0.0035*** (0.0013)	−0.0018* (0.0010)	0.0004 (0.0016)	/	0.0031* (0.0018)	−0.0019** (0.0010)	0.0022 (0.0025)	/
unemp			/								
manu					0.0455*** (0.0119)						
serv							/				
ps									-0.0157 (0.0102)		
hes											/
Control Variables	YES	YES	/	YES	YES	YES	/	YES	YES	YES	/
City fixed effect	YES	YES	/	YES	YES	YES	/	YES	YES	YES	/
Year fixed effect	YES	YES	/	YES	YES	YES	/	YES	YES	YES	/
Observations	1686	1686	/	1686	1686	1686	/	1686	1686	1686	/
R2	0.9934	0.6912	/	0.9656	0.9936	0.9395	/	0.9149	0.9934	0.8393	/

Note: *, **, *** denote significance at the 10%, 5%, and 1% levels, respectively. The values in parentheses are cluster-robust standard errors at the city level.

**Table 7:** Regression results: transmission channels analysis II.

Variables	res (1)	Consumption		Investment		Industrial diversification		Unrelated diversification		Related diversification	
		cons (2)	res (3)	inv (4)	res (5)	div (6)	res (7)	uv (8)	res (9)	rv (10)	res (11)
cov	-0.0020^**^ (0.0009)	−0.1322^**^ (0.0654)	−0.0015^*^ (0.0008)	−0.0316^***^ (0.0093)	−0.0017^*^ (0.0009)	−0.0004 (0.0024)	/	−0.0046^**^ (0.0020)	−0.0019^**^ (0.0010)	0.0041 (0.0030)	/
cons			0.0039^***^ (0.0007)								
inv					0.0099^***^ (0.0025)						
div							/				
uv									0.0154^***^ (0.0046)		
rv											/
Control Variables	YES	YES	YES	YES	YES	YES	/	YES	YES	YES	/
City fixed effect	YES	YES	YES	YES	YES	YES	/	YES	YES	YES	/
Year fixed effect	YES	YES	YES	YES	YES	YES	/	YES	YES	YES	/
Observations	1686	1686	1686	1686	1686	1686	/	1686	1686	1686	/
R^2^	0.9934	0.8984	0.9938	0.2388	0.9935	0.8831	/	0.8311	0.9934	0.9022	/

Note: *, **, *** denote significance at the 10%, 5%, and 1% levels, respectively. The values in parentheses are cluster-robust standard errors at the city level.

The results in Columns (1)-(3) of Table 6 indicate no significant adverse impact of the pandemic on total employment. This finding can be attributed to China’s “employment stabilization policies” during the pandemic [69], as well as to the heterogeneous impacts of COVID-19 across sectors. Regarding employment structure, Columns (4)-(5) reveal that the pandemic significantly reduced the manufacturing employment share, thereby undermining urban economic resilience. Manufacturing-intensive cities are characterized by lower adaptive capacity, which renders them more vulnerable to external shocks [80]. Moreover, global pandemic disruptions substantially impacted manufacturing trade [17], further contracting employment in this sector and adversely affecting urban economic resilience. Columns (6)-(7) indicate that the pandemic’s positive effects on service sector employment were statistically insignificant. Results in Columns (8)-(11) show an increase in producer services employment increased during the pandemic, but no significant growth in high-end services employment. The absence of employment growth in the latter sector thereby limited its potential to bolster urban economic resilience. This phenomenon can be explained by the following factors. As the most dynamic segment within modern service industry system, producer services are key enablers for enhancing urban economic resilience [81]. Although the employment share in China’s producer services segment increased during COVID-19, a substantial portion of this increase was likely concentrated in flexible employment sectors such as food delivery and logistics distribution. According to statistics, Meituan added 457,800 new registered income-earning delivery riders in the first quarter of 2020 [82]. In contrast, knowledge and technology intensive high-end services had no substantial employment growth. Thus, the quantitative expansion of employment in producer services segment occurred without a parallel qualitative upgrading, which limited its role as the significant enabler for enhancing urban economic resilience.

In Table 7, Columns (1)-(3) indicate that the pandemic affected urban economic resilience through consumption channel. Columns (1), (4) and (5) show the impact of COVID-19 on urban economic resilience through investment channel. Column (6) reveals that the shock did not have a statistically significant effect on industrial diversification. We further decompose industrial diversification into unrelated and related diversification. The results in Columns (1), (8) and (9) indicate that unrelated diversification served as one of the channels through which COVID-19 affected urban economic resilience. Meanwhile, the results in Column (10) shows that COVID-19 did not exert a significant negative impact on related diversification. Research suggests that promoting recovery after the pandemic requires not only optimizing industrial production capacity but also actively focusing on regional production conversion capacity. Cities with high technological relatedness density typically demonstrate stronger adaptive capacity. This enables them to swiftly develop substitute industries, thereby dispersing risks, stabilizing employment, and enhancing economic resilience [83]. Li and Hu (2023) argued that related diversification significantly bolsters urban economic resistance in the period following the pandemic, while unrelated diversification exhibits a negative long-term effect under the shock [17]. Based on the regression results, this study shows during COVID-19, industrial diversification ceased to function as an economic stabilizer. This is because unrelated diversification hindered inter-industry collaboration and coordinated adjustment, thereby failing to absorb the shock and constraining the enhancement of urban economic resilience. Overall, COVID-19 impaired urban economic resilience through four channels: employment structure, consumption, investment, and unrelated diversification.

### 4.5 Robustness checks

In robustness check, we first re-estimate the model using an alternative measure of the dependent variable. Specifically, we recalculate urban economic resilience using the entropy-weighted TOPSIS method based on the indicator system in Table 1, and the calculation process of this method can be found in S2 File. This new dependent variable, denoted by *res2*, is then substituted for the original one in Eq (1)-(3). The corresponding results are reported in Table 8, which are consistent with those in Tables 6 and 7 in terms of the signs, magnitudes, and statistical significance of the coefficients. This consistency confirms the robustness of both the baseline regression and transmission channels analysis. To focus the robustness checks on the key significant relationships, we excluded variables whose coefficients for the association with COVID-19 were statistically insignificant, including aggregate employment, service employment, high-end services employment, industrial diversification, and related diversification.

**Table 8:** Robustness test I.

Variables	res2 (1)	manufacturing employment		Producer services employment		Consumption		Investment		Unrelated diversification	
		manu (2)	res2 (3)	ps (4)	res2 (5)	cons (6)	res2 (7)	inv (8)	res2 (9)	uv (10)	res2 (11)
cov	-0.0021^**^ (0.0006)	−0.0035^***^ (0.0013)	−0.0019^***^ (0.0005)	0.0031^*^ (0.0018)	−0.0020^***^ (0.0006)	−0.1322^**^ (0.0654)	−0.0016^***^ (0.0005)	−0.0316^***^ (0.0093)	−0.0019^***^ (0.0005)	−0.0046^**^ (0.0020)	−0.0020^***^ (0.0006)
manu			0.0458^***^ (0.0155)								
ps					-0.0220 (0.0135)						
cons							0.0037^***^ (0.0009)				
inv									0.0051^*^ (0.0031)		
uv											0.0116^*^ (0.0066)
Control Variables	YES	YES	YES	YES	YES	YES	YES	YES	YES	YES	YES
City fixed effect	YES	YES	YES	YES	YES	YES	YES	YES	YES	YES	YES
Year fixed effect	YES	YES	YES	YES	YES	YES	YES	YES	YES	YES	YES
Observations	1686	1686	1686	1686	1686	1686	1686	1686	1686	1686	1686
R^2^	0.9921	0.9656	0.9923	0.9149	0.9922	0.8984	0.9924	0.2388	0.9922	0.8311	0.9922

Note: *, **, *** denote significance at the 10%, 5%, and 1% levels, respectively. The values in parentheses are cluster-robust standard errors at the city level.

To avoid spurious findings in transmission channels analysis, we employed the Sobel test to assess the statistical significance of the mediating effects. Although the three-step approach provides an intuitive analysis of the pandemic’s transmission logic, the risk of spurious findings cannot be entirely ruled out. In contrast, the Sobel test directly computes indirect effect sizes and tests statistical significance by constructing joint standard errors, thereby further ensuring the reliability of the transmission channels analysis. In Table 9, Columns (3)-(4) report the results of Sobel test. The indirect effect sizes through the consumption and investment channels are −0.0005 and −0.0003, accounting for 26.2% and 15.8% of the total effect, respectively. Among all channels, consumption accounts for the largest share of the total effect, underscoring the profound shock that COVID-19 inflicted upon China’s consumer market. During the pandemic, consumption was suppressed by a decline in income and structural unemployment, along with the resultant decreases in shopping frequency and offline spending [39]. This led to a chain reaction of inadequate investment and production, which ultimately adversely affected urban economic resilience.

**Table 9:** Robustness test II.

	manu (1)	ps (2)	cons (3)	inv (4)	uv (5)
Sobel Z	−0.0002^**^ Z = −2.0700	−0.0000 Z = −1.0330	−0.0005^*^ Z = −1.7340	−0.0003^**^ Z = −2.3730	−0.0001^*^ Z = −1.7200
The effect size of mediating variables	−0.0002^**^ Z = −2.0700	−0.0000 Z = −1.0330	−0.0005^*^ Z = −1.73400	−0.0003^**^ Z = −2.3730	−0.0001^*^ Z = −1.7200
The contribution of mediating variables to the total effect	8.1%	/	26.3%	15.8%	3.6%

Note: *, **, *** denote significance at the 10%, 5%, and 1% levels, respectively. The values in parentheses are z-value.

### 4.6 Heterogeneity analysis

As outlined in the literature review, industrial structure is a significant factor influencing urban economic resilience. Academia remains divided on how different types of industrial agglomeration affect urban economic resilience. To investigate the impacts of COVID-19 on urban economic resilience under different industrial agglomeration characteristics, this study conducts subgroup regressions based on three traits: manufacturing specialization, services specialization, and related diversification. Following the approach of An and Huang (2024) [10] and Song et al. (2018) [84], we computed manufacturing and services specialization indices for each city and classified them into high, medium, and low groups.

In Table 10, Columns (1)-(3) display regression results for cities grouped by their level of manufacturing specialization. The results indicate that the economic resilience of cities in both the high and low manufacturing specialization groups was more adversely affected by COVID-19 than that of cities in the medium group. Through its positive economic externalities, manufacturing specialization agglomeration enhances industrial competitiveness, thereby fostering regional economic growth [85]. However, if this kind of agglomeration exceeds a critical threshold, it induces congestion effects in product and factor markets as well as resource environments. These effects trigger homogeneous competition, resource misallocation, and pollution. When the negative externalities of manufacturing specialization agglomeration outweigh benefits, urban economic resilience is undermined [86]. Our findings align with the conclusions mentioned above. COVID-19 exerted significant negative impacts on economic resilience of cities at both ends of manufacturing specialization spectrum.

**Table 10:** Heterogeneity Analysis.

Variables	Manufacturing specialization,			Services specialization			Related diversification		
	Low (1)	Moderate (2)	High (3)	Low (4)	Moderate (5)	High (6)	Low (7)	Moderate (8)	High (9)
cov	-0.0023^**^ (0.0010)	−0.0008 (0.0008)	−0.0025^**^ (0.0012)	0.0039^***^ (0.0006)	−0.0350^**^ (0.0175)	−0.0006 (0.0007)	−0.0229^**^ (0.0107)	−0.0105^***^ (0.0031)	−0.0018^**^ (0.0009)
Control Variables	YES	YES	YES	YES	YES	YES	YES	YES	YES
City fixed effect	YES	YES	YES	YES	YES	YES	YES	YES	YES
Year fixed effect	YES	YES	YES	YES	YES	YES	YES	YES	YES
Observations	540	531	546	537	529	546	588	582	516
R^2^	0.9950	0.9939	0.9944	0.9940	0.9901	0.9945	0.9917	0.9866	0.9952

Note: *, **, *** denote significance at the 10%, 5%, and 1% levels, respectively. The values in parentheses are cluster-robust standard errors at the city level.

In Table 10, Columns (4)-(6) present regression results for cities grouped by their level of services specialization. The economic resilience of cities in the high services specialization group was barely affected by COVID-19. In contrast, the economic resilience of cities in the medium group was more adversely affected by COVID-19 than that of cities in the low group. Cities with a high degree of services specialization not only attract population agglomeration and create abundant jobs, but also amplify inter-industrial technology spillovers and scale effects [87]. Consequently, the impact of COVID-19 on the economic resilience of these cities was negligible. Cities with a medium degree of services specialization exhibit weaker scale effects than those with a high degree of specialization, they still maintain a discernible level of population concentration. However, because COVID-19 initially impacted sectors reliant on face-to-face interaction and dense physical proximity, the economic resilience of cities with a medium degree of services specialization was more adversely affected by COVID-19 than that of cities with a low degree of specialization.

In Table 10, Columns (7)-(9) show regression results for cities grouped by their level of related diversification. The adverse impact of COVID-19 on the economic resilience of cities decreases with increasing related diversification, a finding that is consistent with the results of transmission channels analysis. During the pandemic, a higher degree of related diversification enabled cities to implement government support policies more efficiently and accelerate production conversion as well as product innovation [17], which strengthened their capacity to cope with the shock. Therefore, the economic resilience of cities with a higher degree of related diversification was less adversely affected by COVID-19.

## 5 Discussion

This study measures the economic resilience of 281 Chinese cities at the prefecture-level and above from 2017 to 2022 using the entropy method, and empirically examines the impact of COVID-19 on urban economic resilience and its sub-dimensions by employing two-way fixed effects model. It also analyzes multiple transmission channels through which COVID-19 affects urban economic resilience from supply, demand, and industrial perspectives. Our findings are as follows: (1) COVID-19 significantly impaired overall urban economic resilience. Specifically, it exerted an insignificant negative impact on the sub-dimension of shock resistance, a significant negative impact on the sub-dimension of adaptive recovery, while a positive impact on the innovative transformation dimension. However, innovations compelled by adverse shocks are often subject to time lag, which may account for the statistically insignificant coefficient for the association between COVID-19 and the innovative transformation dimension. These findings remain robust through endogeneity tests. (2) Impact channels analysis reveals that COVID-19 impaired urban economic resilience through four transmission channels: employment structure (primarily via manufacturing employment contraction), consumption, investment, and unrelated diversification. Among these, the consumption channel accounts for the largest share of the total effect, underscoring the profound shock that COVID-19 inflicted upon China’s consumer market. (3) Heterogeneity analysis reveals: For manufacturing specialization, the economic resilience of cities in both the high and low manufacturing specialization groups was more adversely affected by COVID-19 than that of cities in the medium group. For services specialization, the economic resilience of cities in the high services specialization group was barely affected by COVID-19. In contrast, the economic resilience of cities in the medium group was more adversely affected by COVID-19 than that of cities in the low group. Furthermore, the economic resilience of cities with a higher degree of related diversification was less negatively affected by COVID-19.

### 5.1 Theoretical contributions

First, this study extends the literature on urban economic resilience by introducing the context of a global pandemic. Unlike the financial crises typically examined in prior literature, the COVID-19 pandemic was a global public health shock that differs critically from all previous shocks in both scale and nature [17]. This divergence may lead to distinct manifestations of urban economic resilience. Our research updates the shock scenario, and constructs a multidimensional urban economic resilience indicator system grounded in foundational concepts and dynamic evolutionary characteristics. It analyzes the adaptive performance of urban economic systems under a major public health shock across different dimensions, thereby filling a gap in the existing literature regarding the role of crisis attributes in shaping economic resilience.

Second, some scholars used qualitative methods or conducted short-term empirical analyses around 2020, predominantly examining the pandemic’s transmission channels from either supply-side or demand-side perspectives in isolation. However, existing studies haven’t systematically explored the transmission channels through which COVID-19 affects urban economic resilience across different strata of economic system. They have largely oversimplified the determinants of economic resilience and lack a city-level focus in their geographical analysis. Our study addresses these issues by leveraging a comprehensive time frame to analyze potential transmission channels of COVID-19 on urban economic resilience from supply, demand, and industrial perspectives. It examines the pandemic’s effects through total employment, employment structure, consumption, investment, and industrial diversification channels. The study reveals that industrial diversification ceased to function as an economic stabilizer during COVID-19. By decomposing it into related and unrelated diversification, we identified the reason: Unrelated diversification hindered inter-industry collaboration and coordinated adjustment, thereby constraining the enhancement of urban economic resilience after the shock. In summary, this study complements the literature by providing a systematic analysis of transmission channels through which COVID-19 affected urban economic resilience.

Third, given that industrial structure is a significant determinant of urban economic resilience and the effects of different industrial agglomeration types on economic resilience remain widely debated, our study adopts a novel dimension of heterogeneity. Unlike previous studies based on locatio or city size, we conduct subgroup regressions based on three agglomeration traits: manufacturing specialization, services specialization, and related diversification. This allows us to examine the heterogeneous impacts of COVID-19 on urban economic resilience under different industrial agglomeration characteristics. The results indicate that the impact of COVID-19 on urban economic resilience differed significantly across cities grouped by their levels of manufacturing and services specialization. Meanwhile, the results of cities grouped by their level of related diversification are consistent with the findings of the transmission channel analysis. These findings above provide more detailed evidence for understanding the heterogeneous impacts of COVID-19 on urban economic resilience under different industrial agglomeration characteristics.

### 5.2 Policy recommendations

Based on the findings, our study makes the following recommendations. First, policymakers should recognize the challenges and opportunities created by the pandemic: it posed a clear challenge to adaptive recovery while creating an unexpected opportunity for long-term innovative transformation. Thus, policymakers should focus not only on enhancing cities’ capacity to resist external shocks but, more critically, on building their capacities for post-shock recovery and adjustment. Recommended measures include establishing robust emergency response mechanisms, leveraging effective markets and proactive governance to stabilize expectations among firms and residents, thereby boosting market confidence and accelerating economic recovery. Furthermore, as every major shock contains opportunities, policymakers should strategically guide the innovative transformation of urban economies. This can be achieved by identifying and promoting context-specific technological and institutional pathways for each city, thereby accelerating the diffusion of novel technologies and business models, ultimately catalyzing the transformation of urban economic systems.

Second, our findings reveal that employment structure, consumption, investment, and unrelated industrial diversification acted as key channels through which COVID-19 affected urban economic resilience. Regarding employment, local governments should provide short-term support to sectors severely affected by COVID-19 and implement policies to reduce costs for distressed enterprises in order to minimize involuntary layoffs. Concurrently, policymakers should establish training programs aligned with market demand, provide essential employment protections, and facilitate cross-sector redeployment for workers displaced from adversely affected industries. Regarding consumption, after securing employment and stabilizing household incomes, policymakers should work to ensure adequate supply, revitalize traditional consumption, and cultivate new forms of consumption to fully release its potential. Regarding investment, local governments should improve the management of market expectations, guide investment decisions rationally, and foster stable investor confidence. This involves enhancing legal safeguards and establishing public-private cooperation frameworks to cultivate a favorable environment for private investment. Additionally, local governments should boost direct funding for R&D and innovation, while fostering a conducive environment for investments in intangible capital. Regarding industry structure, excessively or indiscriminately diversified economies may be less effective in absorbing public health shocks like COVID-19. Local governments should develop region-specific industrial systems that balance productivity with flexibility to enhance urban economic resilience. This strategy entails formulating industrial diversification policies coherent with local development goals, fostering competitive and diversified industrial systems, and leveraging technological relatedness to drive industrial and product innovation.

Finally, policymakers should tailor policy interventions to the needs of cities with varying levels of specialization in manufacturing and services. For manufacturing, cities below the critical specialization agglomeration threshold should focus on enhancing agglomeration quality while avoiding excessive concentration that leads to congestion effects. In cities that have exceeded this threshold, obsolete and low-tech enterprises must be phased out through a combination of market mechanisms and government regulations, thereby reallocating resources toward more productive industries. For services, policymakers should aim not only to elevate the level of specialization but also to prioritize its qualitative upgrading, thereby fully harnessing the positive externalities of services specialization agglomeration.

## 6 Conclusion

Drawing on panel data analysis encompassing 281 Chinese cities from 2017 to 2022, this study finds that COVID-19 significantly impaired overall urban economic resilience. Specifically, it exerted an insignificant negative impact on the sub-dimension of shock resistance, a significant negative impact on the sub-dimension of adaptive recovery, while a positive impact on the innovative transformation dimension. Furthermore, by examining transmission channels from supply, demand, and industrial perspectives, we find that COVID-19 impaired urban economic resilience through four transmission channels: employment structure (primarily via manufacturing employment contraction), consumption, investment, and unrelated industrial diversification. Among these, the consumption channel accounts for the largest share of the total effect, underscoring the profound shock that COVID-19 inflicted upon China’s consumer market. Situated within the context of COVID-19, our study examines the pandemic’s impact on China’s urban economic resilience and analyzes transmission channels of this impact, drawing on city-level data to provide new empirical insights. Despite its contributions, our study has several limitations. Firstly, the impact of COVID-19 on innovative transformation is not explored in depth due to space constraints. Future studies could examine the pandemic’s impact on innovation through targeted research designs. Secondly, our study focuses exclusively on COVID-19. Given that urban economic systems exhibit different responses and recovery cycles to different types of shocks, our analytical framework excludes other perturbations, such as wars, climate disasters, and trade frictions. Consequently, further studies could comparatively examine responses of urban economic resilience to different shocks to clarify the specific resilience profile across cities.

## Supporting information

S1 File S1 dataset. (XLSX)

S2 File S2 entropy-weighted TOPSIS method. (DOCX)
