# Supplementary material for: Channels and countermeasures of the COVID-19 pandemic’s impact on urban economic resilience: Lessons from China
Source: PLoS One. 2025 Dec 12;20(12):e0338499. doi: 10.1371/journal.pone.0338499 (PMC12700398; doi:10.1371/journal.pone.0338499)
Supplement: S2 File — (DOCX) [file pone.0338499.s002.docx]

**S2 The Entropy-weighted TOPSIS Method**

In the baseline regression part (Section 4.2) of this study, urban economic resilience was calculated by using the entropy method. To mitigate potential weighting bias, we recalculated urban economic resilience with the entropy-weighted TOPSIS method for robustness checks (Section 4.5). The advantage of entropy-weighted TOPSIS lies in its integration of objective weighting with multi-attribute decision-making, unifying solution ranking and comprehensive evaluation. This method applies objective weights obtained via the entropy method to weigh the standardized decision matrix, generating a weighted standardized matrix that reflects indicator importance. Subsequently, the positive ideal solution (comprising optimal indicator values) and negative ideal solution (comprising worst indicator values) are determined based on each indicator’s directional attribute. Euclidean distance formulas then measure geometric distances between evaluation objects and these ideal solutions. Relative closeness serves as the comprehensive evaluation metric, where values closer to 1 indicate proximity to an ideal state. By dynamically assessing solutions’ closeness to optimal/worst benchmarks, this approach enhances result interpretability and decision guidance. Steps to calculation are as follows:

Step one: it is to construct a normalized decision matrix.

$v_{tnm}=\text{W}_{\text{m}}*X_{\text{t}\text{nm}}$；

Step two: it is to determine the positive or negative ideal solutions.

$$V_{tm}^{+}=\left\{ \left( {max}_{n}V_{nm} | m\in positive variables \right),\left( {min}_{n}V_{nm} | m\in\mathrm{negative}\mathrm{variables} \right) \right\}$$

$$V_{tm}^{-}=\left\{ \left( {min}_{n}V_{nm} | m\in positive variables \right),\left( {max}_{n}V_{nm} | m\in\mathrm{negative}\mathrm{variables} \right) \right\}$$

Step three: it is to calculate the Euclidean distances.

$D_{nt}^{+}=\sqrt{\sum_{m=1}^{M} {(v_{tnm}-V_{tm}^{+})}^{2}}$ (positive ideal distance)

$D_{nt}^{-}=\sqrt{\sum_{m=1}^{M} {(v_{tnm}-V_{tm}^{-})}^{2}}$ (negative ideal distance)

Step four: it is to calculate the relative closeness coefficient.​​ Higher values indicate optimal performance.

$C_{tn}=\frac{D_{nt}^{-}}{D_{nt}^{-}+D_{nt}^{+}}$.
